# Vasculature–function relationship in open-angle glaucomatous eyes with a choroidal microvasculature dropout

**DOI:** 10.1038/s41598-022-23109-9

**Published:** 2022-11-14

**Authors:** Anna Lee, Joong Won Shin, Jin Yeong Lee, Min Su Baek, Michael S. Kook

**Affiliations:** Department of Ophthalmology, College of Medicine, Asan Medical Center, University of Ulsan, 88, Olympic-ro 43-gil, Songpa-gu, Seoul, 05505 South Korea

**Keywords:** Diseases, Medical research

## Abstract

Identifying biomarkers associated with functional impairment is important in monitoring glaucoma patients. This retrospective cross-sectional study investigated the vasculature–function relationship in open-angle glaucoma (OAG) eyes with choroidal microvasculature dropout (CMvD) versus in OAG eyes without. Optical coherence tomography (OCT) angiography-derived circumpapillary (cpVD) and macular vessel densities (mVD) were measured in 159 early-stage OAG eyes (mean deviation >  −6 dB) in accordance with the presence or not of a CMvD. OCT-derived circumpapillary retinal nerve fibre layer thickness (cpRNFLT) and macular ganglion cell-inner plexiform layer thicknesses (mGCIPLT) were also measured as reference standards. The vasculature (cpVD and mVD)–function [24-2 visual field mean sensitivity (VFMS) and central 10° VFMS (cVFMS)] and structure (cpRNFLT and mGCIPLT)–function (24-2 VFMS and cVFMS) relationships were compared using global and sectoral maps between OAG eyes with (CMvD+) and without CMvD (CMvD−). The CMvD+ eyes showed significantly steeper cpVD-24-2 VFMS and mVD-cVFMS correlations (P < 0.05). In contrast, there were no significant differences in the cpRNFLT-24-2 VFMS and mGCIPLT-cVFMS relationships between the two groups (P > 0.05). In conclusion, OAG eyes with a CMvD have significantly stronger vasculature–function relationships than eyes without. Vessel density parameters may be useful biomarkers of disease progression in early-stage OAG patients with a CMvD.

## Introduction

Vessel density (VD) assessments using optical coherence tomography angiography (OCT-A) may be an objective method for discriminating glaucomatous patients from healthy subjects, and for monitoring disease progression, since the circumpapillary (cpVD) and/or macular VD (mVD) loss has been observed in eyes with open-angle glaucoma (OAG)^1^, and cpVD and mVD values are strongly associated with the corresponding visual field mean sensitivity (VFMS)^2,3^. Further to this, the strength of cpVD- or mVD-VFMS association is significantly greater than that between the circumpapillary retinal nerve fibre layer (cpRNFLT)- or macular ganglion cell-inner plexiform layer thickness (mGCIPLT)-VFMS association in OAG eyes with moderate to advanced glaucoma^2,3^.

Choroidal microvasculature dropout (CMvD), which is choroidal vascular insufficiency within the β- parapapillary atrophy (β-PPA), is frequently detected in eyes with glaucomatous optic nerve head (ONH) damage^4,5^. At present, it is unclear whether CMvD is a primary event that precedes glaucomatous damage, or a by-product/epiphenomenon of nerve tissue damage in association with glaucoma. Since the parapapillary choroid is closely related to ONH perfusion due to its proximity to, and a common blood supply via the short posterior ciliary arteries^6–8^, and has extensive interconnected networks of microvasculature throughout the structure, CMvD may indicate a sign of global or generalized perfusion insufficiency to the choroid/ONH in glaucomatous eyes^9^. Consequently, OAG eyes with CMvD have more widespread glaucomatous damage and a poorer prognosis compared to those without CMvD at a similar stage of disease severity^10,11^.

In our present study, we hypothesized that the presence of CMvD may have a differential impact on the vasculature–function relationship in glaucomatous eyes, considering that OAG eyes with CMvD (CMvD+ eyes) exhibit greater microvasculature insufficiency in the ONH and choroid compared to OAG eyes without CMvD (CMvD− eyes)^10^. The aim of our present study therefore was to investigate the global and sectoral correlations between the VD measurements and the corresponding VFMS data assessed by standard automated perimetry (SAP), and also to compare the vasculature–function relationships between CMvD+ and CMvD− eyes, matched for disease severity at early-stage glaucoma. We also performed global and sectoral comparisons of the structure–function relationships between the two groups as reference standards.

## Methods

### Study subjects

This retrospective study was approved by the Institutional Review Board (IRB) of Asan Medical Center and adhered to the tenets of the Declaration of Helsinki (Approval code: 2018-1008). The requirement for informed consent from the study subjects was waived by the IRB of Asan Medical Center due to the retrospective nature of the analyses. We consecutively reviewed medical records of patients who visited our glaucoma clinic between April 2018 and November 2021. Each had undergone a comprehensive ophthalmic examination, including best-corrected visual acuity (BCVA), an intraocular pressure (IOP), slit-lamp biomicroscopy, a refractive error determination using an autorefractor (KR-890; Topcon, Tokyo, Japan), axial length (AL, IOL Master version 5; Carl Zeiss Meditec, Dublin, CA), ultrasound pachymetry (Tomey SP-3000, Nagoya, Japan) for central corneal thickness (CCT), dilated colour fundus photography (Canon, Tokyo, Japan), optic disc stereoscopic photography, red-free retinal nerve fibre layer (RNFL) photography (Canon), Humphrey field analyser Swedish Interactive Threshold Algorithm (SITA)-Standard 24-2 VF testing (Carl Zeiss Meditec), spectra-domain OCT (SD-OCT, Cirrus HD; Carl Zeiss Meditec), and OCT-A (AngioVue; Optovue, Inc., Fremont, CA).

The following inclusion criteria were applied to our study subjects: (1) a diagnosis of OAG with a normal anterior chamber and open angles on gonioscopy; (2) age $\ge$ 18 years; (3) BCVA $\ge$ 20/30; (4) spherical equivalent of between −6.0 and + 3.0 dioptres (D) and a cylinder correction within $\pm$ 3.0 D; (5) reliable VF testing (false-positive errors < 15%, false-negative errors < 15%, and fixation loss < 20%). OAG patients were required to satisfy the following specific criteria: (1) the presence of a glaucomatous ONH change (i.e., focal or generalized loss of neural rim and RNFL); (2) glaucomatous VF defects on at least two consecutive tests defined by Andersons’ criteria^12^ irrespective of the IOP level; and (3) a VF mean deviation (MD) better than −6 decibels (dB) for the purpose of evaluating early-stage OAG eyes^12^ to avoid the confounding effects of disease severity on vasculature– and structure–function relationship and the presence of CMvD (i.e., high coexistence of CMvD and advanced glaucoma)^2–4,10^. To account for the learning effects with initial VF testing, a second VF test was performed within 1 week to be used in our final analysis. Subjects with any ophthalmic or neurological disease which may affect the ONH, macular structure, or VF testing were excluded. If both eyes were eligible in any subject, one eye was randomly selected for the study.

### SD-OCT imaging

SD-OCT imaging was performed using Cirrus HD SD-OCT software, version 10.0. The global (360°) cpRNFLT was measured in a 3.46 diameter circle centred on the ONH. The sectoral cpRNFLT was also measured at six sectors according to the Garway-Heath map^13^: temporal (T, 316°–45°), superotemporal (ST, 46°–90°), superonasal (SN, 91°–135°), nasal (N, 136°–225°), inferonasal (IN, 226°–270°), and inferotemporal (IT, 271°–315°) sectors. The cpRNFLT of each sector was estimated by integrating the clock hour RNFL thickness from the Cirrus OCT^14^. The global mGCIPLT was measured at the macular region, centred on the fovea within an annulus with inner vertical and horizontal diameters of 1 and 1.2 mm, and outer vertical and horizontal diameters of 4 and 4.8 mm, respectively. The sectoral mGCIPLT was also measured at six sectors [ST, superocenter (SC), SN, IN, inferocenter (IC), IT].

### OCT-A imaging

OCT-A imaging of the circumpapillary and macular regions was performed using the AngioVue OCT-A system version 8.0 (Optovue Inc) to assure consistency of the data among all of the enrolled study subjects. cpVD measurements were made using images of 4.5 $\times$ 4.5-mm^2^ scans, centred on the optic disc within the radial peripapillary capillary slab from the internal limiting membrane to the nerve fibre layer after the automated removal of large retinal vessels. The OCT-A software automatically provides the cpVD of the peripapillary regions, divided into eight 45° sectors [i.e., ST, SN, IT, IN, temporal upper (TU), temporal lower (TL), nasal upper (NU) and nasal lower (NL)]. To obtain average VD of six sectors in accordance with the Garway-Heath map^13^, temporal (T) and nasal (N) sectors were estimated by averaging of the TU and TL for the T sector and NU and NL for the N sector^2^.

mVD measurements were calculated from a 6 $\times$ 6-mm^2^ region centred on the fovea within the slabs from the internal limiting membrane to the posterior boundary of the inner plexiform layer (i.e., the superficial vascular plexus). Eligible images were then imported into a computer program written using MATLAB software (The MathWorks, Natick, MA) and mVD values were calculated in the same sectors as the mGCIPLT measurements, obtained by Cirrus HD SD-OCT^3^. All SD-OCT and OCT-A images were reviewed by two glaucoma specialists (A.L. and J.Y.L.) for an evaluation of their quality. Images were excluded if they had (1) poor image quality with signal strength < 7; (2) motion artifacts; (3) localized weak signal intensity caused by vitreous floaters or poor clarity (i.e., media opacity); (4) images with fixation error; or (5) segmentation failure^9–11^.

### Choroidal microvasculature dropout assessment

The CMvD within the $\upbeta$-PPA zone was evaluated on en face images of choroidal layer from the retinal pigment epithelium to 390 $\upmu$m below the Bruch membrane^15^. CMvD was defined as a localized complete loss of the choriocapillaries and choroidal microvasculature within the $\upbeta$-PPA, and identified when the minimum angular width was greater than the width of the central retinal vein^4,10,16,17^. The margins of the optic disc, β-PPA and CMvD were evaluated by two glaucoma specialist (A.L. and J.Y.L.), while being blind to the clinical, VF, and SD-OCT information of the study patients. The presence of CMvD was confirmed regardless of its multiplicity (n $>$ 1) or location (superior vs. inferior) within the β-PPA zone. Following the initial enrolment of OAG patients, each case was consecutively classified into two groups based on the presence of CMvD (i.e., CMvD + vs. CMvD− group).

### Definition of central 10$^\circ$ visual field loss and mapping the vasculature (cpVD and mVD) and structure (cpRNFLT and mGCIPL) to function (SAP) correlations

**Figure 1:**
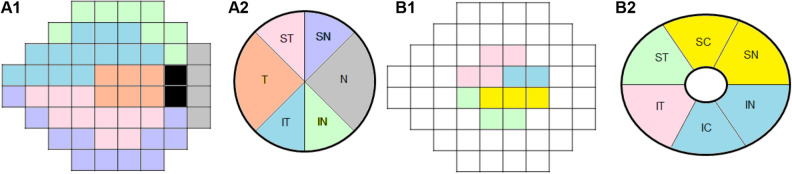
Vasculature– and structure–function correspondence map: visual field (VF) sectors (**A1**) and corresponding circumpapillary vessel density and retinal nerve fiber layer thickness sectors (**A2**). VF sectors within the central 10$^\circ$ VF area (**B1**) and corresponding macular vessel density and ganglion cell-inner plexiform layer thickness sectors (**B2**). *IC* inferocenter, *IN* inferonasal, *IT* inferotemporal, *N* nasal, *SC* superocenter, *ST* superotemporal, *SN* superonasal, *T* temporal, *VF* visual field.

In the current study, central 10° VF loss was defined as clusters of three points in the central 10° with P < 0.05 on the pattern deviation map, or up to two significant points in central 10° with P < 0.01, regardless of VF damage extension to the 10–24° VF area^18^. Vasculature– and structure–function relationships were defined as correlation coefficients between VD parameters and thickness parameters, and the corresponding VFMS values, according to the regionalization described by Garway-Heath et al.^13^. The VFMS was calculated by switching from a logarithmic dB to a nonlogarithmic dB [10 × log(1/Lambert);1/L scale]^18,19^. The global 24-2 VFMS was calculated as the mean of VF sensitivities in 52 points of the SAP, except 2 points just above and below the blind spot and grouped into six VF sectors: SN, N, IN, IT, T, and ST (Fig. 1A1,A2)^13^. In addition, the central VFMS (cVFMS) in the 12 central 10° VF points of the SAP, which corresponds topographically to the macular region within 4.8 mm of the center of the fovea^20^, was calculated and grouped into four central VF sectors (i.e., ST, SN, IN, and IT sectors) topographically according to previous publications (Fig. 1B1,B2)^13,18–20^. Briefly, ST cVFMS was defined as the average sensitivity of two ST VF points (paired with IN + IC mGCIPLT or IN + IC mVD) and SN cVFMS was defined as the average sensitivity of four SN VF points (paired with IT mGCIPL or IT mVD). IN cVFMS was defined as the average sensitivity of three IN VF points (paired with ST mGCIPLT or ST mVD) and IT cVFMS was defined as the average sensitivity of three IT VF points (paired with SN + SC mGCIPLT and SN + SC mVD). These six 24-2 and four central 10° VF sectors were correlated with corresponding regional cpRNFLT and cpVD (Fig. 1A1,A2) and mGCIPLT and mVD measurements (Fig. 1B1,B2), respectively^13,18–20^.

**Figure 2:**
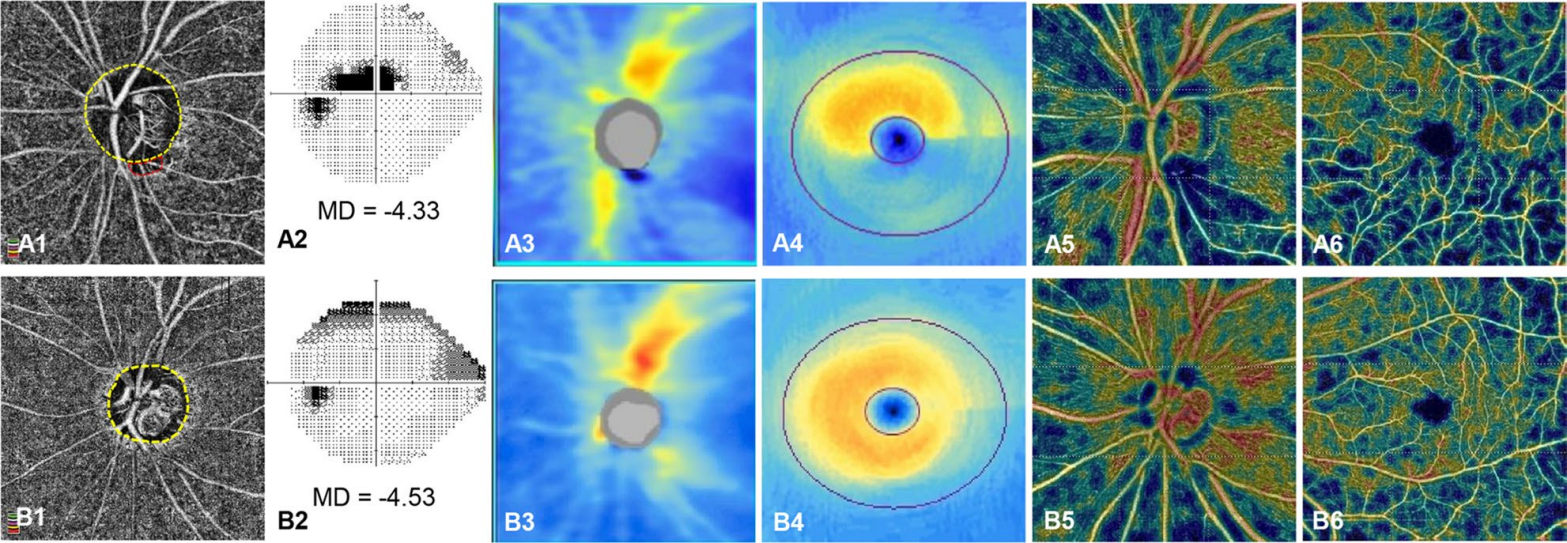
Representative cases (**A,B**) of open-angle glaucoma (OAG) eyes with and without choroidal microvasculature dropout (CMvD) showing different clinical characteristics. In the En face parapapillary choroidal layer image from optical coherence tomography angiography (OCT-A) analysis of OAG eye with CMvD (**A1**) and that of OAG eye without CMvD (**B1**), yellow and red dot outlines indicate the optic nerve head and CMvD borders, respectively. The CMvD+ eye showed a central visual field (VF) defect (**A2**) with a VF mean deviation (MD) of −4.33 decibel (dB), whereas the CMvD− eye showed a peripheral VF defect (**B2**) with a similar VF MD of −4.53 dB. On the spectral-domain optical coherence tomography (SD-OCT) thickness map, the CMvD+ eye showed localized circumpapillary retinal nerve fibre layer thickness (cpRNFLT) loss at the inferior temporal (IT) sector (50.5 $\mu \mathrm{m})$ and at the temporal (T) sector (58.0 $\mu \mathrm{m}),$ which was adjacent to the site of the CMvD (**A3**). The CMvD+ eye also revealed macular ganglion cell-inner plexiform layer (mGCIPL) thinning at the inferior hemiretina, including IT sector (57.3 $\mu \mathrm{m}$, **A4**). On the color-coded OCT-A map, the circumpapillary vessel density (cpVD) reduction was detected at the IT region (23.7%, **A5**), while a macular vessel density (mVD) reduction (36.4%) was also observed at the inferior hemiretina (**A6**). The CMvD− case (**B1**), however, demonstrated a peripheral VF defect (**B2**) with cpRNFLT loss at the IT sector (61.0 $\mu \mathrm{m}$, **B3**), while there was less pronounced mGCIPL thinning at this sector (70.0 $\mu \mathrm{m}$, **B4**). On the color-coded OCT-A map, despite the slight cpVD reduction detected at the IT region (39.7%, **B5**) where the cpRNFLT loss was observed, an mVD reduction in the CMvD− eye was not as apparent in the inferior hemiretina (**B6**) as that in the CMvD+ case.

### Statistical analysis

All statistical analyses were performed using SPSS version 21.0 (IBM Corp, Chicago, IL) and SAS version 9.4 (SAS Institute Inc., Cary, NC). P values less than 0.05 were considered statistically significant and results were presented as either mean values with a standard deviation or as a frequency and percentage. The inter-observer agreement for the presence and location of the CMvD was assessed using the kappa (k) statistic. The normality of distribution was assessed using the Kolmogorov–Smirnov test. Normally distributed data were compared using an independent Student t test; otherwise, Mann–Whitney U tests were used. Categorical variables were compared using chi-square tests between the groups. The correlations of vasculature– and structure–function relationships were evaluated using linear regression analysis^18,19^. In all of the regression analyses, the VFMS was regarded as the dependent variable and the cpRNFLT, mGCIPLT, cpVD and mVD values as independent variables. To compare correlation coefficients for vasculature– and structure–function between the groups (CMvD+ vs. CMvD− group), the bootstrapping method with a repetition of 1000 times with replacement was performed to examine for statistically significant differences between two correlation coefficients^21^. Each r value was derived from 1000 bootstrapped data and the difference between r values of the two groups was then calculated. Assuming that the bootstrapped values of the difference followed a normal distribution, a t-test was performed to test the null hypothesis that their r means between two groups were equal^21^. Clinical variables associated with 24-2 and central 10° VFMS in each OAG group were analysed using univariable and multivariable linear regression analysis. Variables with P values < 0.1 in the univariable analysis were included in the multivariable linear regression analyses with a backward elimination process.

## Results

Of the 181 OAG eyes of 181 patients who were initially enrolled in our present study cohort, 22 eyes with unreliable VF testing and poor-quality SD-OCT/OCT-A scans were excluded. Hence, our final cohort comprised 159 eyes, consisting of 67 CMvD+ eyes and 92 CMvD− eyes. The inter-observer agreements for the determination of the presence and location of CMvD were excellent. (k = 0.935, 0.911, respectively). The demographics and baseline characteristics are summarized in Table 1. In CMvD+ eyes, CMvD was most frequently observed in the inferior hemiretina (52 eyes, 77.6%). There was no significant difference in the mean VF MD values between the groups (−3.73 dB vs. −3.74 dB; P = 0.960). However, the CMvD+ eyes had significantly lower global mGCIPLT, cpVD and mVD values (P < 0.05). With respect to VF findings, the CMvD+ eyes showed significantly higher prevalence of superior hemifield VF defects and central 10$^\circ$ VF loss (P < 0.05). Consequently, cVFMS values of the CMvD+ eyes were significantly lower than those of the CMvD− eyes (P = 0.007). In the sectoral comparison of OCT and OCT-A parameters (Table 2), the CMvD+ eyes showed significantly lower IT sector of cpRNFLT, lower inferior (IT, IC and IN) and SN sectors of mGCIPLT, lower T and IT sectors of cpVD, and lower six sectors of mVD compared to the CMvD− eyes (P < 0.05). Regarding the sectoral VFMS map (Table 2), the CMvD+ eyes showed significantly lower 24-2 VFMS values at N sector and lower central 10° VFMS values at ST and SN sectors compared to the CMvD− eyes (P < 0.05).

**Table 1:** Comparison of the baseline characteristics between open-angle glaucoma eyes with and without choroidal microvasculature dropout.

	CMvD+ (n = 67)	CMvD− (n = 92)	P-value
Age, year	60.1 $\pm$ 13.2	57.2 $\pm$ 11.8	0.152
Sex, male/female	30:37	46:46	0.518
Laterality, right/left	31:36	38:54	0.939
Hypertension, n (%)	14 (20.9)	17 (18.5)	0.672
Diabetic mellitus, n (%)	3 (4.5)	11 (11.9)	0.085
Central corneal thickness, $\it \upmu$m	535.69 $\pm$ 34.06	536.27 $\pm$ 33.45	0.921
Spherical equivalent, diopter	−2.09 $\pm$ 2.63	−1.39 $\pm$ 2.65	0.114
Axial length, mm	24.48 $\pm$ 1.36	24.31 $\pm$ 1.20	0.462
Intraocular pressure, mmHg	13.1 $\pm$ 2.0	14.0 $\pm$ 2.4	**0.013**
Number of glaucoma medications, n	1.3 $\pm$ 0.9	0.9 $\pm$ 0.9	**0.010**
Disc area, mm^2^	1.99 $\pm$ 0.44	1.90 $\pm$ 0.40	0.340
Rim area, mm^2^	0.80 $\pm$ 0.16	0.83 $\pm$ 0.18	0.184
CMvD location, n (%)			
Superior	7 (10.5)	–	–
Inferior	52 (77.6)	–	–
Both	8 (11.9)	–	–
Optic disc haemorrhage, n (%)	10 (14.9)	9 (9.8)	0.402
VF mean deviation, dB	–3.73 $\pm$ 1.31	–3.74 $\pm$ 1.55	0.960
VF defect location, n (%)			
Superior	46 (68.7)	42 (45.7)	**0.004**
Inferior	11 (16.4)	29 (31.5)	**0.030**
Both	10 (14.9)	21 (22.8)	0.214
Central 10$^\circ$ VF loss, n (%)	60 (89.5)	52 (56.5)	**<0.001**
24-2 VFMS, 1/L	828.14 $\pm$ 255.34	776.63 $\pm$ 243.04	0.198
Central 10$^\circ$ VFMS, 1/L	1032.22 $\pm$ 394.23	1208.29 $\pm$ 402.12	**0.007**
Global cpRNFLT, $\it \upmu$m	72.95 $\pm$ 9.47	73.41 $\pm$ 9.29	0.763
Global mGCIPLT, $\it \upmu$m	66.85 $\pm$ 7.28	72.49 $\pm$ 6.14	**<0.001**
Global cpVD, %	43.52 $\pm$ 4.95	45.28 $\pm$ 4.33	**0.020**
Global mVD, %	41.20 $\pm$ 4.92	45.10 $\pm$ 5.94	**<0.001**

*CMvD* choroidal microvasculature dropout, *cpRNFLT* circumpapillary retinal nerve fibre layer thickness, *cpVD* circumpapillary vessel density, *dB* decibel, *L* Lambert, *mGCIPLT* macular ganglion cell-inner plexiform layer thickness, *MS* mean sensitivity, *mVD* macular vessel density, *VF* visual field, *VFMS* visual field mean sensitivity.

Significant values are in bold.

**Table 2:** Sectoral quantitative comparison of OCT/OCT-A and VFMS data between open-angle glaucoma eyes with and without choroidal microvasculature dropout.

	CMvD+ (n = 67)	CMvD− (n = 92)	P-value
**24-2 VFMS, 1/L**			
ST VF sector	578.04 $\pm$ 289.69	493.71 $\pm$ 301.96	0.079
T VF sector	934.73 $\pm$ 426.94	831.38 $\pm$ 379.57	0.110
IT VF sector	912.35 $\pm$ 362.26	725.72 $\pm$ 411.39	**0.003**
IN VF sector	1120.68 $\pm$ 507.89	907.93 $\pm$ 469.28	**0.007**
N VF sector	1155.05 $\pm$ 565.42	1457.31 $\pm$ 531.88	**0.001**
SN VF sector	502.09 $\pm$ 332.06	561.80 $\pm$ 341.88	0.273
**Central 10**$^\circ$** VFMS, 1/L**			
ST VF sector	853.88 $\pm$ 702.24	1307.05 $\pm$ 536.95	**<0.001**
SN VF sector	538.91 $\pm$ 502.28	888.91 $\pm$ 533.63	**<0.001**
IN VF sector	1290.21 $\pm$ 617.52	1137.41 $\pm$ 607.26	0.122
IT VF sector	1550.85 $\pm$ 699.53	1639.17 $\pm$ 581.73	0.387
**Global cpRNFLT, **$\it {\varvec{\upmu}}$**m**			
ST sector	84.62 $\pm$ 20.14	78.74 $\pm$ 21.92	0.089
T sector	58.52 $\pm$ 11.27	60.74 $\pm$ 60.74	0.233
IT sector	62.51 $\pm$ 18.83	71.96 $\pm$ 18.86	**0.002**
IN sector	67.51 $\pm$ 12.65	66.34 $\pm$ 11.83	0.552
N sector	62.26 $\pm$ 8.90	60.38 $\pm$ 9.84	0.221
SN sector	79.87 $\pm$ 12.39	79.86 $\pm$ 12.89	0.995
**Global mGCIPLT, **$\it {\varvec{\upmu}}$**m**			
ST sector	69.03 $\pm$ 10.42	71.42 $\pm$ 8.56	0.116
IT sector	57.47 $\pm$ 9.78	66.71 $\pm$ 8.51	**<0.001**
IC sector	58.64 $\pm$ 9.53	69.20 $\pm$ 7.94	**<0.001**
IN sector	68.10 $\pm$ 9.97	76.68 $\pm$ 6.87	**<0.001**
SN sector	75.91 $\pm$ 9.65	79.10 $\pm$ 8.19	**0.026**
SC sector	71.51 $\pm$ 11.38	74.32 $\pm$ 9.75	0.097
**Global cpVD, %**			
ST sector	45.67 $\pm$ 10.38	44.23 $\pm$ 10.78	0.404
T sector	49.78 $\pm$ 5.89	53.33 $\pm$ 3.67	**<0.001**
IT sector	30.05 $\pm$ 10.64	39.95 $\pm$ 11.53	**<0.001**
IN sector	41.29 $\pm$ 8.62	41.92 $\pm$ 9.12	0.659
N sector	42.95 $\pm$ 6.57	43.28 $\pm$ 5.71	0.732
SN sector	43.97 $\pm$ 7.49	42.17 $\pm$ 8.78	0.179
**Global mVD, %**			
ST sector	41.77 $\pm$ 5.62	44.50 $\pm$ 6.51	**0.008**
IT sector	36.75 $\pm$ 5.90	42.07 $\pm$ 6.73	**<0.001**
IC sector	39.04 $\pm$ 6.16	45.12 $\pm$ 6.82	**<0.001**
IN sector	43.03 $\pm$ 5.01	46.89 $\pm$ 5.98	**<0.001**
SN sector	43.05 $\pm$ 5.08	45.67 $\pm$ 6.57	**0.009**
SC sector	43.27 $\pm$ 5.16	46.46 $\pm$ 5.92	**0.001**

*CMvD* choroidal microvasculature dropout, *cpRNFLT* circumpapillary retinal nerve fibre layer thickness, *cpVD* circumpapillary vessel density, *IC* inferocenter, *IN* inferonasal, *IT* inferotemporal, *L* Lambert, *mGCIPLT* macular ganglion cell-inner plexiform layer thickness, *MS* mean sensitivity, *mVD* macular vessel density, *N* nasal, *OCT* optical coherence tomography, *OCT-A* optical coherence tomography angiography, *SC* superocenter, *SN* superonasal, *ST* superotemporal, *T* temporal, *VF* visual field, *VFMS* visual field mean sensitivity.

Significant values are in bold.

Comparisons of vasculature– and structure–function relationships between the groups are presented in Table 3. In the assessment of vasculature–function relationships, the CMvD+ eyes showed significantly stronger global and sectoral cpVD–VFMS relationship at nasal VF sector in the 24-2 map corresponding to T sector of cpVD and cpRNFLT (P < 0.05). In the central 10° VF area, the CMvD+ eyes showed significantly stronger global and sectoral mVD–cVFMS relationship at ST and SN VF sectors corresponding to IT and IC + IN sectors of mVD and mGCIPLT (P < 0.05). Regarding structure–function relationships, however, there were no significant differences in the cpRNFLT–VFMS relationships in the 24-2 map or in the mGCIPLT–cVFMS relationships in the central 10° VF map, either globally or in any sector, between the two groups (P > 0.05).

**Table 3:** Comparison of the vasculature– and structure–function relationships between open-angle glaucoma eyes with and without choroidal microvasculature dropout.

	cpVD-VFMS			cpRNFLT-VFMS		
	CMvD+ (n = 67)	CMvD− (n = 92)	P-value	CMvD+ (n = 67)	CMvD− (n = 92)	P-value
24-2 map, global	**0.624 (<0.001)**	**0.376 (<0.001)**	**0.015**	**0.502 (<0.001)**	**0.474 (<0.001)**	0.812
ST VF sector	**0.527 (<0.001)**	**0.512 (<0.001)**	0.894	0.179 (0.150)	**0.320 (0.002)**	0.345
T VF sector	**0.370 (0.002)**	**0.342 (0.001)**	0.825	0.001 (0.997)	**0.212 (0.042)**	0.163
IT VF sector	**0.523 (<0.001)**	**0.453 (<0.001)**	0.467	**0.349 (0.004)**	**0.321 (0.002)**	0.834
IN VF sector	**0.714 (<0.001)**	**0.629 (<0.001)**	0.230	**0.594 (<0.001)**	**0.706 (<0.001)**	0.204
N VF sector	**0.546 (<0.001)**	**0.276 (0.008)**	**0.026**	**0.453 (<0.001)**	**0.394 (<0.001)**	0.659
SN VF sector	**0.761 (<0.001)**	**0.573 (<0.001)**	0.059	**0.760 (<0.001)**	**0.609 (<0.001)**	0.164

	mVD− cVFMS			mGCIPLT-cVFMS		
	CMvD+ (n = 67)	CMvD− (n = 92)	P-value	CMvD+ (n = 67)	CMvD− (n = 92)	P-value
Central 10$^\circ$ VF map, global	**0.643 (<0.001)**	**0.373 (<0.001)**	**0.032**	**0.505 (<0.001)**	**0.415 (<0.001)**	0.466
ST VF sector	**0.609 (<0.001)**	**0.295 (<0.001)**	**0.012**	**0.615 (<0.001)**	**0.386 (<0.001)**	0.060
SN VF sector	**0.655 (<0.001)**	**0.318 (0.002)**	**0.021**	**0.640 (<0.001)**	**0.602 (<0.001)**	0.757
IN VF sector	**0.394 (<0.001)**	**0.251 (0.016)**	0.291	**0.522 (<0.001)**	**0.607 (<0.001)**	0.367
IT VF sector	**0.471 (<0.001)**	**0.401 (<0.001)**	0.587	**0.307 (0.011)**	**0.289 (0.003)**	0.892

*CMvD* choroidal microvasculature dropout, *cpRNFLT* circumpapillary retinal nerve fibre layer thickness, *cpVD* circumpapillary vessel density, *IC* inferocenter, *IN* inferonasal, *IT* inferotemporal, *L* Lambert, *mGCIPLT* macular ganglion cell-inner plexiform layer thickness, *MS* mean sensitivity, *mVD* macular vessel density, *N* nasal, *OCT* optical coherence tomography, *OCT-A* optical coherence tomography angiography, *SC* superocentre, *SN* superonasal, *ST* superotemporal, *T* temporal, *VF* visual field, *VFMS* visual field mean sensitivity.

Significant values are in bold.

Table 4 present the results of the univariable and multivariable linear regression analysis conducted to determine the clinical factors associated with global VFMS in the 24-2 and central 10$^\circ$ VF map of each OAG group. In multivariable analysis, age and the global cpVD were significantly associated with the global VFMS in the 24-2 map of the CMvD+ eyes, while age and the global cpRNFLT showed this relationship in the CMvD− eyes. In the central 10$^\circ$ VF map, age and the global mGCIPLT and mVD were the factors found to be significantly associated with central 10$^\circ$ VFMS in the CMvD+ eyes, while age and mGCIPLT had this association in the CMvD− eyes. Figure 2 shows representative CMvD+ and CMvD− cases from our OAG study cohort, illustrating the stronger association between the VFMS loss and cpVD/mVD reduction in the CMvD+ eye, as opposed to weaker association between VFMS loss and cpVD/mVD reduction in the CMvD− eye.

**Table 4:** Univariate and multivariate linear regression analyses to determine the clinical variables associated with global 24–2 and central 10$^\circ$ visual field mean sensitivity in open-angle glaucoma eyes with and without choroidal microvasculature dropout.

Global 24-2 VFMS	CMvD+						CMvD−					
	Univariable model			Multivariable model			Univariable model			Multivariable model		
	β	95% CI	P-value	ß	95% CI	P-value	β	95% CI	P-value	β	95% CI	P-value
Age, year	−11.743	−15.573 to −7.922	**<0.001**	−6.159	−11.108 to −1.210	**0.016**	−11.905	−15.407 to −8.402	**<0.001**	−11.288	−14.772 to −7.805	**<0.001**
IOP, mmHg	24.119	−6.127 to 45.365	0.116				1.331	−20.085 to 22.748	0.902			
CCT, $\mathrm{\mu m}$	−0.794	−2.737 to 1.149	0.417				1.674	0.103 to 3.245	**0.037**			
Axial length. mm	−28.331	−0.203 to 96.864	0.051				33.809	−14.548 to 82.166	0.168			
ODH	19.130	−10.806 to 146.323	0.765				3.180	−101.684 to 108.045	0.952			
Rim area, mm^2^	516.745	140.737 to 892.753	**0.008**				226.053	−43.864 to 495.971	0.100			
Disc area, mm^2^	14.707	−127.888 to 157.302	0.8337				−97.694	−232.766 to 37.377	0.154			
cpRNFLT, $\mathrm{\mu m}$	13.564	7.676 to 19.452	**<0.001**				12.411	7.586 to 17.236	**<0.001**	9.974	5.828 to 14.120	**<0.001**
cpVD, %	32.320	22.050 to 42.590	**<0.001**	19.990	3.958 to 36.021	**0.016**	20.435	9.897 to 30.974	**<0.001**			
**Central 10**$^\circ$** VFMS**												
Age, year	−14.773	−21.225 to −8.321	**<0.001**	−9.191	−14.876 to −3.506	**0.002**	−19.588	−25.400 to −13.777	**<0.001**	−18.592	−25.087 to −12.098	**<0.001**
IOP, mmHg	42.286	−4.148 to 88.720	0.074				5.388	−30.033 to 40.808	0.302			
CCT, $\mathrm{\mu m}$	−0.892	−3.920 to 2.136	0.557				1.879	−0.753 to 4.511	0.159			
Axial length. mm	62.210	−17.996 to 142.416	0.125				72.639	−7.537 to 152.815	0.075			
ODH	−12.457	−208.950 to 184.036	0.900				47.382	−125.844 to 220.607	0.588			
Rim area, mm^2^	637.726	45.158 to 1230.294	**0.035**				226.493	−224.433 to 677.419	0.321			
Disc area, mm^2^	−40.194	−260.203 to 179.815	0.716				−94.334	−319.005 to 130.337	0.406			
mGCIPLT, $\mathrm{\mu m}$	27.373	15.795 to 38.950	**<0.001**	17.126	7.473 to 26.778	**0.001**	27.037	14.547 to 39.528	**<0.001**	17.138	3.767 to 30.508	**0.013**
mVD, %	51.416	35.467 to 67.365	**<0.001**	32.172	16.566 to 47.777	**<0.001**	25.281	12.120 to 38.441	**<0.001**			

$\beta$ $\beta$-coefficient, *CCT* central corneal thickness, *CI* confidence interval, *CMvD* choroidal microvasculature dropout, *cpRNFLT* circumpapillary retinal nerve fibre layer thickness, *cpVD* circumpapillary vessel density, *IOP* intraocular pressure, *mGCIPLT* macular ganglion cell-inner plexiform layer thickness, *mVD* macular vessel density, *ODH* optic disc hemorrhage.

Significant values are in bold.

## Discussion

VD reduction as determined by OCT-A is known to be highly associated with the loss of VFMS in glaucoma patients^2,3,22^, which indicates the potential role of VD as an additional surrogate marker to monitor functional loss in glaucoma. As the presence of CMvD is linked to microvasculature insufficiency of the ONH/choroid and a poorer prognostic outcome^4,10,15^, it is plausible to hypothesize that vasculature–function relationships may differ between OAG eyes with and without CMvD. In this current study, OAG eyes with CMvD revealed stronger global and sectoral vasculature–function relationships in the 24-2 and central 10° VF maps, while there were no differences in the structure–function relationships between the two groups. These findings suggest that VD parameters, as measured by OCT-A, may be useful biomarkers for detecting and monitoring glaucomatous damage in OAG eyes, particularly in those showing signs of choroidal microvasculature insufficiency such as CMvD. To our knowledge, this present study is the first to demonstrate different impacts on the vasculature–function relationship in OAG eyes according to the presence of CMvD.

Our current analyses indicated that the global mean cpVD was significantly lower in the CMvD+ eyes but that there was no significant difference in the global cpRNFLT values between the two groups (Table 1). In sectoral assessments, the CMvD+ eyes showed a significantly lower cpRNFLT at the IT sector and lower cpVD at both the IT and T sectors (Table 2). These findings suggest that OAG eyes with CMvD show more severe structural and microvasculature damage than those without CMvD, despite both groups having a similar age, AL, and VF MD. There are several possible explanations for these observations. First, the damaged blood–brain barrier at the choriocapillary non-perfusion area within the β-PPA zone, as represented by CMvD, can promote the release of vasoactive or toxic substances into the ONH, leading to axonal damage, RNFL loss, and cpVD reduction^23,24^. Another possibility is that as the parapapillary choroid is closely linked to ONH perfusion, insufficient blood flow within this structure in the eyes with CMvD can result in diminished blood flow to ONH, thereby leading to deep ONH structural damage, such as LC morphologic changes, which can subsequently induce axonal and vascular loss in the superficial retina^4–8,25^. Of note in this regard, sectoral loss of the cpVD and cpRNFLT was found to be spatially correlated with CMvD location and was mostly observed at the T and/or IT sectors in the CMvD+ eyes in the present study series (83.5%). These findings are in line with those of a previous study, which also reported an association between CMvD and reduced cpVD and RNFLT with topographic correlation^5^, and with prior reports that CMvDs are most often found at the IT region in the optic disc, which is the most vulnerable area to disruption of the microvasculature and ONH structural damage^5,16,26^.

In our present study series, the global and sectoral mean mVD and mGCIPLT values were significantly lower in the CMvD+ eyes, except for the ST and SC sectors of mGCIPLT (Tables 1 and 2). The CMvD+ eyes also showed a significantly higher prevalence of central VF loss as well as a lower cVFMS at the ST and SN sectors compared to CMvD- eyes, despite both groups having similar overall VF MD values (Tables 1 and 2). One speculation in relation to these findings is that since CMvD is most often located near the macular vulnerability zone, which was first described by Hood et al.^27^, its presence can be directly related to the loss of macular structural parameters (i.e., mGCIPLT and mVD) and central VF defects^16,28^. In addition, as the density of retinal ganglion cells (RGCs) is highest in the central macula, the RGCs in the macular region require a high oxygen supply, rendering them particularly vulnerable to microvascular hypo-or non-perfusion conditions such as CMvD.

Apart from the difference in the location of preferred VF damage (i.e., central VF), the CMvD+ eyes showed more widespread macular structure and VD loss compared to the CMvD− eyes in the current study. Despite that CMvD was mostly located only in the inferior hemiretina (77.6%), CMvD+ eyes showed global loss of mGCIPLT and mVD, with mean mGCIPLT and mVD values being significantly lower at the SN sector and ST, SC, and SN sectors (i.e., opposite side of CMvD location), respectively, compared to the CMvD− eyes (Table 2). Our findings have clinical significance that the CMvD+ eyes may have more severe and generalized macular structural and VD loss compared to the CMvD− eyes.

Our comparisons of the vasculature–function relationships between the two groups demonstrated that global and sectoral vasculature–function relationship were significantly stronger in the CMvD+ eyes, according to either the 24-2 or central 10° VF maps (Table 3). One explanation for these observations is that despite both groups having similar VF MD values after matching for glaucoma severity (i.e., early-stage glaucoma), choroidal microvasculature insufficiency in the form of CMvD may induce generalized deficiency of blood flow in the ONH and retina of CMvD+ eyes^9^, thus leading to more pronounced loss of cpVD/mVD and VFMS globally and sectorally^22^. This in turn result in stronger vasculature–function relationships in the CMvD+ eyes based on both 24-2 and central 10° VF maps. Another explanation is that greater severity of disease in the CMvD+ eyes may be related to steeper vasculature–function relationship in these eyes, despite both CMvD+ and CMvD− eyes having similar VF MD. Considering that disruption of superficial microvasculature may be secondary to the axonal loss^4^, VD loss may not be profound enough to reflect functional loss in early stage of glaucoma. In contrast, more severe VD loss observed in the CMvD+ eyes may have contributed to steeper vasculature–function relationship compared to CMvD− eyes.

It is noteworthy that there were no significant differences in the global and sectoral structure–function relationships between the eyes with and without CMvD, according to either the 24-2 or central 10° VF maps (Table 3). Although the explanation for these findings is unclear, one possible reason for this observation is that RGC damage induced by microvasculature insufficiency, as observed in the CMvD+ eyes, might not have completely manifested into sufficient cpRNFLT/mGCIPLT reduction, thus resulting in similar structure–function relationships between the two groups. Munguba et al.^29^ reported that changes in RNFL thickness following crush injury lag behind the decline of RGC function, based on their histopathologic analyses. Hence, although the mechanisms of ONH injury may differ between the two studies, ONH/retinal damage induced by CMvD may first lead to microvasculature loss and functional damage, followed by a slower degeneration of the RNFL, which may explain our present findings that VD loss is better associated with the RGC dysfunction and subsequent VFMS loss than RNFL thinning. Further studies with a longitudinal design are needed to validate our hypothesis in the future.

The linear regression analyses showed that a reduced global cpVD and mVD were independently associated with the corresponding global 24-2 and central 10° VFMS in the CMvD+ eyes, but this association was found with a reduced global cpRNFLT and mGCIPLT in the CMvD− eyes. These findings may further support our speculation that VD parameters have stronger relationship with functional loss in eyes with CMvD. Interestingly, the global cpRNFLT was not significantly associated with global 24-2 VFMS, whereas mGCIPLT was independently associated with cVFMS, in the CMvD+ eyes (Table 4). Marshall et al.^30,31^ recently reported that glaucoma patients with vascular risk factors such as hypertension or myocardial infarction predominantly showed mGCIPLT loss, rather than a cpRNFLT reduction. These authors hypothesized that vascular dysfunction may be important in glaucomatous damage involving the macular structure. Our current findings of a significant association between the cVFMS and mGCIPLT in the CMvD+ eyes may be explained by microvasculature insufficiency present in those eyes.

There were several limitations of note in the present study. First, en face choroidal images of an OCT-A may sometimes have technical limitations in accurately detecting CMvD. For example, projection artifacts, such as shadowing effects from the optic disc hemorrhage (ODH) or projection flow from the superficial retinal layer, can make it difficult to define the presence of CMvD and/or ONH/CMvD boundary. Nonetheless, we attempted to exclude the influence of large overlying retinal vessels and/or ODH on our scanning laser ophthalmoscopy images within the $\beta$-PPA during the assessment of CMvD by having multiple examiners to evaluate the OCT-A images based on a method validated in previous studies^4,9,16,17^. Second, the six sectors covered by the circumpapillary and macular OCT-A/OCT maps may not completely match the corresponding sectoral VFMS sectors topographically. However, these regions are automatically provided by these devices and closely matched with the corresponding VFMS based on the previously validated regionalization map of Garway-Heath et al.^13,18–20^. Third, as we evaluated early-stage OAG eyes in our present analyses, our results may be limited in terms of their generalizability to other patients with different types and severity of glaucoma. However, since the vasculature– and structure–function relationships vary according to disease severity, early-stage OAG eyes were selected to compare the relationship of vasculature/structure with the corresponding VFMS in the two OAG groups, while removing the confounding effects of advanced disease severity on the CMvD. Fourth, we did not evaluate the potential confounding effects of topical IOP-lowering medications on vasculature–function relationships, since ocular hypotensive medications may affect ocular blood flow^32^, thus affecting VD measurements. Hence, our findings should be cautiously interpreted due to the possibility of confounding effects of topical hypotensive medications. Fifth, the homogeneous Korean population analysed in this study may have introduced a selection bias, in which our findings may not fully reflect those of general populations in other countries. Hence, validation of our data is needed in clinically similar populations outside of Korea. Sixth, our cross-sectional study design did not provide information on the temporal relationship between the CMvD and vasculature–function correlations. Lastly, CMvD may not be considered as a phenotype of choroidal microvasculature insufficiency, since the pathogenic mechanism of CMvD is currently undetermined and more scientific evidence is needed. However, based on indocyanine green angiography study, CMvD has been observed as a localized perfusion defect in the parapapillary choroid resulting from occlusion of the choroidal vessels. Thus, CMvD may indicate vascular insufficiency to the ONH structure such as prelaminar or laminar tissue^4,33^. Furthermore, it has been revealed that CMvD was independently associated with potential risk factors of vascular insufficiency, such as a lower mean ocular perfusion pressure, migraine and cold extremity^28^.

In conclusion, early-stage OAG eyes with CMvD show a significantly greater degree of global and regional macular VD loss, and stronger vasculature–function relationships, compared to eyes without CMvD. Our findings suggest that VD parameters derived from an OCT-A may be useful biomarkers of disease progression in early-stage OAG patients with choroidal microvasculature dropout.

## Data Availability

The datasets generated and analyzed during the current study are available from the corresponding author on reasonable request.
