# Author Correction: Vasculature–function relationship in open-angle glaucomatous eyes with a choroidal microvasculature dropout

**DOI:** 10.1038/s41598-023-27967-9

**Published:** 2023-01-17

**Authors:** Anna Lee, Joong Won Shin, Jin Yeong Lee, Min Su Baek, Michael S. Kook

**Affiliations:** Department of Ophthalmology, College of Medicine, Asan Medical Center, University of Ulsan, 88, Olympic‑ro 43‑gil, Songpa‑gu, Seoul, 05505 South Korea

Correction to: *Scientific Reports* 10.1038/s41598-022-23109-9, published online 14 November 2022

The original version of this Article contained an error in Figure 2 panel A1, where the yellow dashed circle was inadvertently duplicated.

The original Figure 2 and accompanying legend appear below.

**Figure 2:**
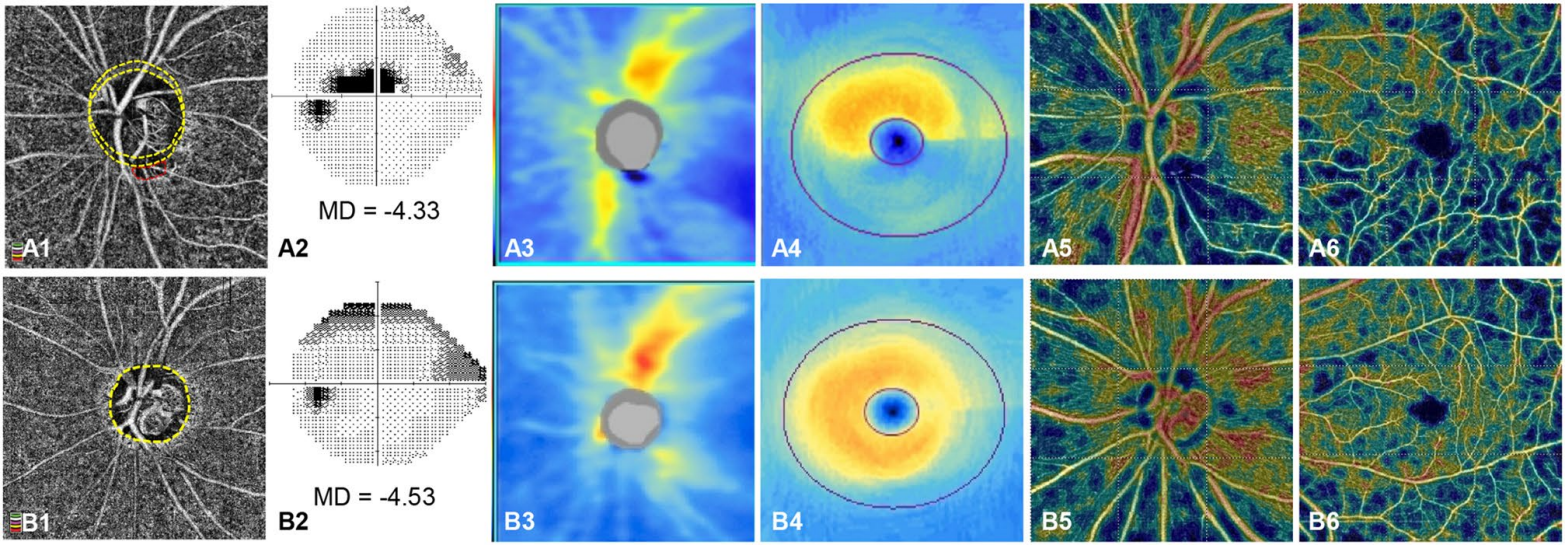
Representative cases (**A,B**) of open-angle glaucoma (OAG) eyes with and without choroidal microvasculature dropout (CMvD) showing different clinical characteristics. In the En face parapapillary choroidal layer image from optical coherence tomography angiography (OCT-A) analysis of OAG eye with CMvD (**A1**) and that of OAG eye without CMvD (**B1**), yellow and red dot outlines indicate the optic nerve head and CMvD borders, respectively. The CMvD+ eye showed a central visual field (VF) defect (**A2**) with a VF mean deviation (MD) of − 4.33 decibel (dB), whereas the CMvD− eye showed a peripheral VF defect (**B2**) with a similar VF MD of − 4.53 dB. On the spectral-domain optical coherence tomography (SD-OCT) thickness map, the CMvD+ eye showed localized circumpapillary retinal nerve fibre layer thickness (cpRNFLT) loss at the inferior temporal (IT) sector (50.5 μm) and at the temporal (T) sector (58.0 μm), which was adjacent to the site of the CMvD (**A3**). The CMvD+ eye also revealed macular ganglion cell-inner plexiform layer (mGCIPL) thinning at the inferior hemiretina, including IT sector (57.3 μm, **A4**). On the color-coded OCT-A map, the circumpapillary vessel density (cpVD) reduction was detected at the IT region (23.7%, **A5**), while a macular vessel density (mVD) reduction (36.4%) was also observed at the inferior hemiretina (**A6**). The CMvD− case (**B1**), however, demonstrated a peripheral VF defect (**B2**) with cpRNFLT loss at the IT sector (61.0 μm, **B3**), while there was less pronounced mGCIPL thinning at this sector (70.0 μm, **B4**). On the color-coded OCT-A map, despite the slight cpVD reduction detected at the IT region (39.7%, **B5**) where the cpRNFLT loss was observed, an mVD reduction in the CMvD− eye was not as apparent in the inferior hemiretina (**B6**) as that in the CMvD+ case.

The original Article has been corrected.

